# Computed tomography evaluation of internal nasal valve angle and area and its correlation with NOSE scale for symptomatic improvement in rhinoplasty^☆^

**DOI:** 10.1016/j.bjorl.2019.08.009

**Published:** 2019-10-03

**Authors:** Amr G. Shafik, Hussam Adel Alkady, Gehad Mohamed Tawfik, Ahmed Mostafa Mohamed, Tahany Mohamed Rabie, Nguyen Tien Huy

**Affiliations:** a Ain Shams University, Faculty of Medicine, Department of Otorhinolaryngology, Cairo, Egypt; b Ain Shams University, Faculty of Medicine, Cairo, Egypt; c Online Research Club (http://www.onlineresearchclub.org/), Japan; d Ain Shams University, Faculty of Medicine, Department of Diagnostic Radiology, Cairo, Egypt; e Duy Tan University, Institute of Research and Development, Da Nang, Vietnam.

**Keywords:** NOSE scores, Rhinoplasty, CT-scan, INV, Area, Angle, Postoperative, Escore NOSE, Rinoplastia, Tomografia computadorizada, VNI, Área, Ângulo, Pós-operatório

## Abstract

**Introduction:**

Nasal obstruction is one of the most frequent otolaryngologic complaints; and the collapse of the internal nasal valve is one the main causes of the nasal air flow obstruction.

**Objective:**

We aimed to evaluate internal nasal valve by using reformatted CT-scans pre- and post- rhinoplasty at 3 months and to assess its correlation to symptomatic improvement of nasal obstruction using the NOSE scale.

**Methods:**

A prospective observational study was conducted between March 2017–May 2018 in a tertiary care otorhinolaryngology center. We included patients suffering from nasal obstruction secondary to internal nasal angle collapse and nasal deformity. Patients with sinusitis, nasal polyposis, and nasal masses were excluded.

**Results:**

Twenty consecutive patients underwent rhinoplasty, with a mean age (22.2 ± 2.8), with majority of males (n = 14; 70%). There was no significant correlation between pre- and post- CT-scans of the internal nasal valve angle/area and NOSE scores. A high significant difference was detected between mean pre- and post- NOSE scores (*p* < 0.0001), which was absent in CT-scan results.

**Conclusion:**

Reformatted CT-scans measurements of internal nasal valve area and angle were not of value. NOSE scores pre- and post- rhinoplasty had a significant value to determine degree of obstructive symptom improvement.

## Resumo

**Introdução:**

A obstrução nasal é uma das queixas otorrinolaringológicas mais comuns, e o colapso ou obstrução da válvula nasal interna é a principal causa da obstrução das vias aéreas nasais.

**Objetivo:**

Objetivamos avaliar a válvula nasal interna pré, e 3 meses pós-rinoplastia utilizando tomografias computadorizadas reformatadas e avaliar sua correlação com a melhora do sintoma obstrutivo utilizando a escala NOSE, do Inglês *Nasal Obstruction Symptom Evaluation*.

**Método:**

Um estudo observacional prospectivo foi realizado entre março de 2017 e maio de 2018 em um centro de otorrinolaringologia de atenção terciária. Foram incluídos pacientes que apresentavam obstrução nasal secundário a colapso da valva nasal interna e deformidade nasal. Pacientes com sinusite, polipose nasal e tumores nasais foram excluídos.

**Resultados:**

Vinte pacientes consecutivos, a maioria do sexo masculino (n = 14; 70%) com média de idade de 22,2 ± 2,8 anos, foram submetidos a rinoplastia eincluídos no estudo. Não houve correlação significante entre as avaliações tomográficas pré / pós cirúrgicas do ângulo/área da valva nasal interna e os escores NOSE. Foi detectada uma diferença altamente significante entre os escores médios da escala NOSE entre o pré e pós-operatório (p < 0,0001), mas não nos resultados da avaliação por tomografia computadorizada.

**Conclusão:**

As medidas reformatadas de tomografias computadorizadas da área e ângulo da valva nasal interna pré e pós cirúrgicas não tiveram valor. Entretanto, os escores da escala NOSE pré e pós-rinoplastia mostraram uma diferença significante ao determinar o grau de melhora do sintoma obstrutivo.

## Introduction

Nasal obstruction is one of the most common complaints of patients in Otorhinolaryngology (ORL) medicine.^1^ Collapse or obstruction of the Internal Nasal Valve (INV) is mostly the cause of nasal airway obstruction.^2^, ^3^ Airflow resistance is very essential during breathing for good pulmonary function. The INV is considered the narrowest part of nasal airway, hence, has the greatest resistance flow.^2^ Fifty percent of total airway resistance is from nasal airway resistance, which mostly occurs in the anterior part of nose which called INV.^4^ Anatomically, the INV is located roughly 1.3 cm from nares and is bordered by septum medially, by upper lateral cartilages laterally, and by anterior end of inferior turbinate inferiorly and nose floor.^5^ It is a slit-like opening; its cross-sectional area is about 55–65 mm.^6^, ^7^, ^8^ In asymptomatic people, The INV angle is about 10°‒15° between upper lateral cartilage and septum.^3^, ^9^, ^10^

The INV is a remarkably significant area for ORL surgeons to correctly evaluate before rhinoplasty and/or septoplasty surgery for patients with nasal obstruction. Different surgical techniques are used to correct INV collapse during functional rhinoplasty, which may include partial inferior turbinectomy, septoplasty, and placement of spreader grafts, which is one of the most effective methods of correcting nasal obstruction.^11^ Despite many studies which have evaluated the functional and anatomical analysis of nasal cavity, a standard accurate measure of nasal obstruction has not yet been verified.^4^, ^12^ No gold standard test is being used nowadays to diagnose INV obstruction.^5^ Many tools have been used in evaluation of nasal resistance and INV, including rhinomanometry and acoustic rhinometry, however, they possess the limitations, of lacking reliability and requiring expensive equipment.^6^, ^7^, ^13^ To overcome these limitations, Computed Tomography (CT) scans and Quality Of Life (QOL) questionnaires have been used to assess implication and outcomes of ORL surgeries for nasal valve collapse. CT-scan has been suggested as an objective tool to measure INV anatomy pre- and post-operatively.^14^, ^15^ However, it was found that using the conventional coronal imaging plane does not result in sufficient evaluation of INV. Cakmak et al.^16^ and Poetker et al.,^17^ suggest that INV angle may be better assessed when CT-scans are reformatted to a plane perpendicular to the estimated acoustic axis.^16^, ^17^ These studies confirm that CT may be a worthy tool in objectively evaluating outcomes of functional nasal surgeries..

Stewart et al. developed and validated the Nose Obstruction Symptom Evaluation (NOSE) scale as a symptomatic improvement quality of life scale, to be used in evaluation of INV obstruction.^1^ The NOSE scale was reported in studies as a useful tool for assessing INV pre- and post- surgeries.^18^, ^19^, ^20^ Proper preoperative evaluation of the INV is critical to the workup decisions developed for repair of this area’s problems. Asking a patient to do a CT scan for INV angle/area preoperative can provide the ORL surgeon with upgraded anatomical information to evaluate the INV.^21^ To fill this void for knowing which is the best evaluation tool to be used before and after rhinoplasty, we uniquely evaluated theINV area and angle using reformatted CT-scans, and thencompared its results to NOSE scores scale for assessing symptomatic improvement of patients undergoing rhinoplasty for nasal obstruction plus nasal deformity pre- and post- rhinoplasty. Targeting the detection of the most effective tools for evaluation of INV before rhinoplasty and for followup after surgery can be instructive.

## Methods

### Patients and data collection

We performed a prospective observational cohort study of twenty consecutive patients from March 2017 till May 2018, who underwent rhinoplasty surgery at the Otorhinolaryngology department in Ain Shams University Hospital, Cairo, Egypt. Institutional Review Board approval was received from Vice Dean of Medical Ethics Committee of Faculty of Medicine Ain Shams University for Graduate Studies and Research. All patients signed informed written consent without receiving any stipend, and they were informed about study requirements according to World Medical Association Declaration of Helsinki (version 2002). All patients agreed to undergo a CT-scan of the nose pre-rhinoplasty and three months post-rhinoplasty. Complete ORL examination and endoscopic assessment of the nose was completed in all patients prior to surgery. Inclusion criteria were as follows: male and female patients with range 20–50 years old, nasal obstruction secondary to internal nasal valve collapse, nasal deformity, and/or inferior turbinate hypertrophy, and/or deviated nasal septum. Exclusion criteria were as follows: sinusitis, nasal polyposis, and nasal masses.

### Measuring tools

**Figure 1:**
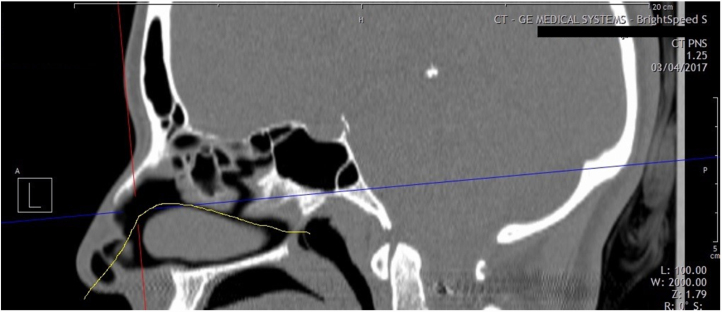
Sagittal reformatted computed tomographic view of nasal cavity. The yellow line indicates the estimated acoustic axis and physiologic nasal airflow.

Reformatted coronal CT-scans of INV area/angle were requested at a plane perpendicular to the anterior aspect of acoustic axis. Acoustic axis is determined on a sagittal reformatted image based on results of Cakmak study,^16^ who revealed that axis passes through the center of nasal passage in an arc (Fig. 1). Functional septorhinoplasty was done with spreader graft insertion in most of the patients.

**Figure 2:**
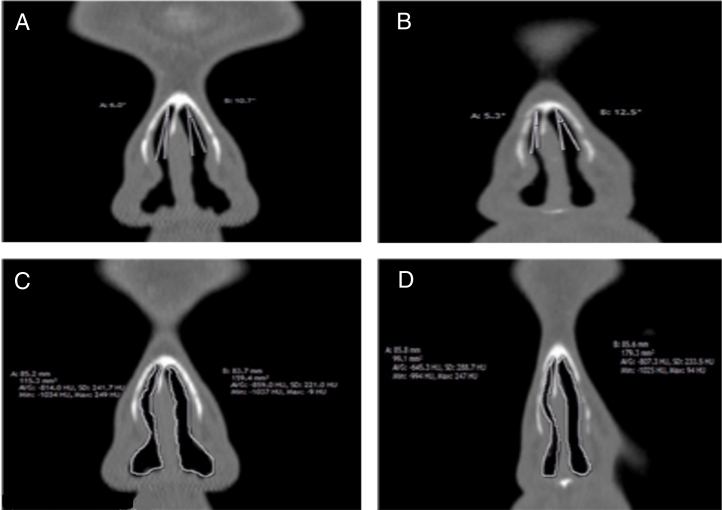
Internal nasal valve angle/area pre and postoperative by reformatted coronal CT-scans. A, angle pre-operative; B; angle post-operative; C, area pre-operative, D, area post-operative.

We measured the INV cross-sectional area and valve angle through a standardized section (1 mm cut, immediately anterior to head of inferior turbinate)^22^ from reformatted scans as shown in one case of our patients (Fig. 2A–D). All patients were asked to complete NOSE scale questionnaire before and 3 months after surgery. Sums of the answers were multiplied by five out of a total score of a 100.^1^, ^19^

### Statistical analysis

The statistician who performed statistical analysis was blinded and uninvolved with clinical record or radiologic analysis. All data were collected, coded, tabulated and analyzed using SPSS version 24.0 software (IBM Corp. Released 2016. IBM SPSS Statistics for Windows, Armonk, NY: IBM Corp).

### Sample characteristics

Data were normally distributed, therefore, mean and Standard Deviation (SD) were calculated for parametric numerical data, while frequency and percentage for non-numerical data. A Shapiro–Wilk’s test (*p*-value > 0.05)^23^ and visual inspection of their normal Q-Q plots and box plots showed that the post nasal scores were approximately normally distributed for both males and females,^24^ with a skewness of 0.591 (Standard Error ‒ SE = 0.597) and a kurtosis of −0.108 (SE = 1.154) for the males and a skewness of 0.857 (SE = 0.845) and a kurtosis of −0.300 (SE = 1.741) for the females (Supplementary Fig. S1).^25^

Pearson correlation coefficient (r) was used to assess strength of association between INV angle and area measures from each nares and average of both sides with NOSE scores measurements to define the strength and direction of the linear relationship between them. Student *t* test was used to assess statistical significance of difference between two study group means. All reported *p*-values originated from two-sided tests with statistical significance defined as *p* < 0.05. Linear regression was done to test and estimate the dependence of a quantitative variable based on its relationship with a set of independent variables.

Stewart et al developed and validated NOSE scale as a disease-specific QOL instrument to be used in nasal obstruction problems (Supplementary Table S1).^1^, ^19^

## Results

Table 1 shows patients’ characteristics (n = 20). A total of 20 patients met our eligibility criteria and underwent rhinoplasty surgery. Of the 20 patients examined, 14 were male (70%) and 6 were female (30%) with ages ranging from 20 to 50 years old and age mean ± SD (22.2 ± 2.8). Two patients (10%) had a history of previous rhinoplasty and 18 (90%) had a history of nasal trauma. All patients (100%) initially complained of nasal obstruction in at least one nares. Follow-up for patients was for three months.

**Table 1:** Demographic, symptomatic, and clinical features of included 20 patients.

Patient code	Age (years)	Sex	Indication for rhinoplasty		Preoperative					Postoperative				
			Post-traumatic (Yes/No)	Revision (Yes/No)	NOSE score	INV area (mm^2^)		INV angle (degree)		NOSE score	INV area (mm^2^)		INV angle (degree)	
						Lt. side	Rt. side	Lt. side	Rt. side		Lt. side	Rt. side	Lt. side	Rt. side
P1	23	F	No	Yes	55	170.1	140.4	11.7	9.1	35	132.2	133.0	12.1	15.0
P2	20	M	Yes	No	75	73.7	230.5	8.7	9.1	45	79.1	101.7	7.0	9.4
P3	24	M	Yes	No	70	108.5	138.2	5.6	7.7	40	118.3	176.6	5.1	10.4
P4	23	M	Yes	No	80	161.5	93.8	13.1	0	40	131.3	178.2	10.0	14.5
P5	21	F	Yes	No	60	72.7	98.2	8.2	12.9	35	150.2	114.5	18.4	16.0
P6	22	M	Yes	No	80	133.2	74.6	12.0	0	45	145.8	131.2	9.5	6.1
P7	21	F	Yes	No	55	159.4	115.3	10.7	6.0	40	179.3	99.1	12.5	5.3
P8	20	M	Yes	No	60	111.5	181.3	20.1	8.9	50	86.2	108.7	11.8	9.4
P9	20	M	Yes	No	65	198.4	127.4	9.3	8.3	40	110.0	184.7	5.9	14.7
P10	23	M	Yes	No	65	251.7	99.7	17.1	5.4	35	129.0	149.9	11.3	11.5
P11	21	F	Yes	No	60	150.9	89.8	13.9	6.4	40	148.2	150.3	5.2	8.3
P12	20	M	Yes	No	70	121.6	302.1	6.0	20.1	35	191.0	158.2	13.6	18.9
P13	20	F	Yes	No	70	107.8	110.9	7.4	8.4	35	143.0	163.6	10.7	12.1
P14	26	M	Yes	No	60	109.9	99.9	9.1	8.0	30	118.7	100.0	10.0	7.4
P15	27	M	Yes	No	65	112.0	174.7	3.7	11.7	30	139.9	106.2	6.9	8.6
P16	21	M	No	Yes	80	100.3	229.3	0	19.5	30	113.9	143.9	12.4	13.9
P17	20	M	Yes	No	75	140.7	154.4	6.5	12.1	55	95.8	111.1	7.8	11.1
P18	20	M	Yes	No	70	80.9	66.9	5.8	8.8	35	108.5	117.4	10.0	7.0
P19	30	F	Yes	No	65	82.3	84.4	9.1	5.4	45	74.1	77.4	8.9	8.6
P20	22	M	Yes	No	70	158.9	161.5	9.0	15.3	40	109.5	114.0	8.0	6.8

INV, internal nasal valve; NOSE, Nose Obstruction Symptom Evaluation; M, male; F, female; P, patient; Lt., left; Rt., right.

N.B, All patients undergo rhinoplasty surgery and evaluated pre and post-surgery by reformatted coronal computed tomography scans and NOSE scale.

Comparing between males and female postoperative nasal scores showed no significant difference between them, where the mean ± SD was 39.3 ± 7.6 and 38.3 ± 4.1 (*p* = 0.78), respectively.

Patient’s symptomatic improvement from nasal obstruction was evaluated using (NOSE) scale and showed that the mean preoperative (NOSE) score was 67.5 ± 7.9 and mean postoperative (NOSE) score was 39 ± 6.6 with a mean change of (NOSE) score 28.5 ± 9.3, indicating a highly statistically significant difference between pre and postoperative NOSE scores (*p* < 0.0001), with the most difference between pre- and post-operative NOSE scores was 50 (80 vs. 30) (Table 2).

**Table 2:** Radiographically measurements of left and right reformatted coronal CT-scans of INV angle and cross-sectional area.

	Preoperative			Postoperative			*p*-Value
	Mean ± SD	Minimum	Maximum	Mean ± SD	Minimum	Maximum	
INV area Lt. Side	130.3 ± 44.9	72.7	251.7	125.2 ± 30.4	74.1	191.0	0.642
INV area Rt. Side	138.7 ± 60.7	66.9	302.1	130.9 ± 30.7	77.4	184.7	0.613
Average INV area	134.5 ± 52.8	73.9	211.9	128.1 ± 30.5	75.8	174.6	0.431
INV angle Lt. Side	9.4 ± 4.6	0	20.1	9.9 ± 3.2	5.1	18.4	0.683
INV angle Rt. Side	9.2 ± 5.2	0	20.1	10.8 ± 3.7	5.3	18.9	0.170
Average INV angle	9.3 ± 4.9	6.0	14.5	10.3 ± 3.5	6.75	17.2	0.121
NOSE score	67.5 ± 7.9	55.0	80.0	39.0 ± 6.6	30.0	55.0	0.0001^a^

INV, internal nasal valve; NOSE, Nose Obstruction Symptom Evaluation; SD, standard deviation; Lt., left; Rt., right.

a *p*-value is significant, *p* < 0.05.

### Radiographically measurements of coronal CT-scans of INV cross-sectional area

The INV cross-sectional area showed that mean preoperative INV area on the left side, right side, and average of both were 130.3 ± 44.9, 138.6 ± 60.6, and 134.5 ± 52.8, respectively. As well, it showed that the mean postoperative INV area on the left side, right side and average of both were 125.2 ± 30.4, 130.9 ± 30.7 and 128.1 ± 30.5, respectively. There was no statistically radiologically significant difference between either means of pre- and post-operative INV area on left, right, or their average (Table 2).

It was found that there is no statistically significant correlation between preoperative NOSE scores and preoperative INV cross-sectional areas either on left side, right side, or average of both with correlation coefficient (r) (r = −0.16; *p* = 0.5), (r = 0.207; *p* = 0.381), (r = 0.077; *p* = 0.746), respectively. There was a statistically significant negative correlation between postoperative NOSE scores and postoperative INV cross-sectional area on left side (r = −0.444; *p* = 0.05) (Table 3 and Supplementary Fig. S2).

**Table 3:** Pearson correlation for association of INV angle/area measurements with postoperative NOSE scores.

	Preoperative		Postoperative	
	Correlation coefficient	*p*-Value	Correlation coefficient	*p*-Value
INV area Lt. Side	−0.160	0.500	−0.444	0.050^a^
INV area Rt. Side	0.207	0.381	−0.197	0.405
Average INV area	0.077	0.746	−0.394	0.085
INV angle Lt. Side	−0.367	0.111	−0.282	0.228
INV angle Rt. Side	0.021	0.930	−0.225	0.341
Average INV angle	−0.347	0.133	−0.298	0.201

INV, internal nasal valve; NOSE, Nose Obstruction Symptom Evaluation; Lt., left; Rt., right.

a *p*-value is significant, *p* < 0.05.

Otherwise, no statistically significant correlation was detected on right side (r = −0.197; *p* = 0.405) or average of both sides (r = −0.394; *p* = 0.085) (Table 3).

### Radiographically measurements of coronal CT-scans of INV angle

The INV angle showed that mean preoperative INV angle on the left side, right side, and average of both were 9.4 ± 4.6, 9.15 ± 5.2 and 9.3 ± 4.9, respectively. As well, it showed that the mean postoperative INV angle on the left side, right side and average of both were 9.9 ± 3.2, 10.8 ± 3.7 and 10.3 ± 3.5, respectively. Results revealed that there was no statistically radiologically significant difference between either means of pre- and post-operative INV area on left, right, or their average (Table 2).

Preoperatively, there was no statistically significant correlation between preoperative NOSE scores and preoperative INV angles either on left side, right side, or average of both (r = −0.367; *p* = 0.111), (r = 0.021; *p* = 0.93), (r = −0.347; *p* = 0.133), respectively. Postoperatively there was no statistically significant correlation between postoperative NOSE scores and postoperative INV angles either on left side, right side, or an average of both (r = −0.282; *p* = 0.228), (r = −0.225; *p* = 0.341), (r = −0.298; *p* = 0.201), respectively (Table 3).

In correlation between cases age and postoperative NOSE scores, there was no statistically significant correlation detected (r = −0.206; *p* = 0.384).

After adjustment to all factors, age, sex, postoperative INV area and angle, multiple regression revealed that none of the factors was statistically affecting postoperative NOSE scores with regression coefficient (−1.035, *p* = 0.081), (0.76, *p* = 0.81), (−0.12, *p* = 0.079), (−0.556, *p* = 0.312), respectively (Table 4).

**Table 4:** Multiple regressions to study independent factors affecting postoperative NOSE scores.

Factors	Regression coefficient	*p*-Value	95%CI
Age	−1.035	0.081	0.146, -0.215
Sex	0.760	0.810	−5.750, 7.270
Post INV area	−0.120	0.079	−0.250, 0.015
Post INV angle	−0.556	0.312	−1.690, 0.576

INV, internal nasal valve.

## Discussion

There was a highly significant difference between the mean of pre- and pos-toperative NOSE scores. The highest mean change of NOSE scores was 50, while lowest mean change was 10 postoperatively. No significant difference was found between the mean of pre- and post-operative radiological measurements of cross-sectional area and angle of INV. In addition, no correlation was found between radiological CT-scans of area/angle and NOSE score measurements.

Many physiologic and psychological factors can affect patients’ perception of nasal obstruction and outcomes of surgeries. Those factors include: expectations of patients from surgery outcomes, different surgical techniques, and status of vascular and nerve supplies.^26^ The coincidence of sinusitis or any nasal or sinuses masses with nasal obstruction is statistically linked to higher rates of dissatisfaction after surgery, which may give wrong impressions on effectiveness of measurable method used. For that our exclusion criteria were: sinusitis, nasal polyposis, and nasal masses that affect airway resistance and give the wrong impression that surgery does not give good results quickly as expected, as those exclusions need additional time for feeling improvement with a mandatory continuation on medical treatment.^27^ This is a decisive judgment on the success of rhinoplasty surgery from a scientific and legal view. Surgeons in ORL need a reliable method to prove their indications for surgery are proper, or for followup of patients by assessing degree of obstruction pre- and post-surgery. Although no accurate method has been validated yet, the NOSE scale was found to be a promising and reliable method to assess symptomatic improvement of nasal obstruction problems. A study has been proposed for the efficacy of CT-scan for INV assessment.^17^ Previously they recommended using CT-scan planes to be in perpendicular line to acoustic axis, however, most institutions do not have availability for employing complex reformatting planes. Moreover, most radiologists use standard images as being more familiar for them. When they applied axial planes correctly, a more adequate visualization of INV ensued, incorporating all three bordering structures.^17^ Kahveci et al.^28^ reported that mean preoperative NOSE scores were 60.2 ± 17.5 and mean postoperative score was 11.3 ± 10.5, with minimum and maximum NOSE scores at 20 and 80. In our study, we found that there was no statistically significant difference between pre- and postoperative INV cross-sectional areas, either on left side, right side or the average of both sides. For these results, CTs can radiological measurements must not give accurate an impression of patient symptomatic improvement after surgery. As regards pre- and postoperative INV angle/area, there was no statistically significant difference either on the left side, right side or the average of both sides. In accordance with our findings, Veron et al. confirmed that CT scan analysis of INV does not produce an objective evaluation of the degree of nasal o bstruction; it is better to be correlated with septal morphology as a possible cause of nasal obstruction symptoms.^29^ In addition, Bloom et al., confirmed the importance of taking in consideration the preoperative physical examination, and not just CT imaging in the decision for which patients are candidates forsurgical intervention.^22^ In contrast, Moche et al.^30^ reported that radiographic imaging of INV area produced good sensitivity and specificity values, making it a good measurement tool for INV assessment.

On the other hand, after evaluation of symptomatic improvement according to NOSE scores scale, there was a highly statistically significant difference between pre- and postoperative NOSE scores, with significant improvement in the scores after surgery, which agrees with the results of many studies.^19^, ^28^, ^31^, ^32^, ^33^, ^34^, ^35^ A collective systematic review was conducted collecting all included NOSE papers scores published until 2014, revealing that NOSE scores can be used as a clinically significant measure of successful surgical outcomes.^36^ Even Ishii et al. detected that NOSE scores could be used as a screening tool instead of the Epworth sleepiness scale for patients at risk of undiagnosed obstructive sleep apnea.^37^

As well, there was no significant correlation between CT scans postoperative INV angles and areas with postoperative (NOSE) score. Thus, there was no correlation between CT scans analysis of the INV and the (NOSE) scale, which agrees with Veron et al.^29^ Kahveci et al.,^28^ who detected a significant negative correlation between angle degree increase and NOSE scores. Our results beg a question of whether physical examination findings by NOSE scores scale should be indicated as a mandatory measurement tool over postoperative radiologic assessments for follow-up, to be used in concordance with CT-scans preoperative for more accurate surgical outcome plans.

In spite of our attempts to discover an objective measurement, the study of 20 patients as a sample size may not serve as a true normal population. However, patients who had any significant nasal and sinus disorders that could affect nasal obstruction average improvement time were excluded.

## Conclusion

Radiological measurements of cross-sectional area and angle of INV by reformatted CT scans were not of value in evaluation of INV pre and post-rhinoplasty. On the contrary, there was a highly significant difference between NOSE scores pre and post-rhinoplasty, in which it was found to be of significant value to determine the degree of symptomatic improvement pre and post rhinoplasty. Therefore for better clinical symptomatic evaluation pre- and post-operative in rhinoplasty; using NOSE scale assessment is preferable to using routine CT scan commonly requested by ORL surgeons for some decision making after physical examination. Besides, NOSE scale would be a useful follow-up measurement tool for assessment of the degree of symptomatic improvement.

## Conflicts of interest

The authors declare no conflicts of interest.
