# Supplementary material for: Computed tomography evaluation of internal nasal valve angle and area and its correlation with NOSE scale for symptomatic improvement in rhinoplasty
Source: Braz J Otorhinolaryngol. 2019 Oct 3;86(3):343–50. doi: 10.1016/j.bjorl.2019.08.009 (PMC9422510; doi:10.1016/j.bjorl.2019.08.009)
Supplement: Supplementary file 1 [file mmc1.docx]

Supplementary

Supplementary Figure 1. Tests of normality of normal Q-Q and box plots.


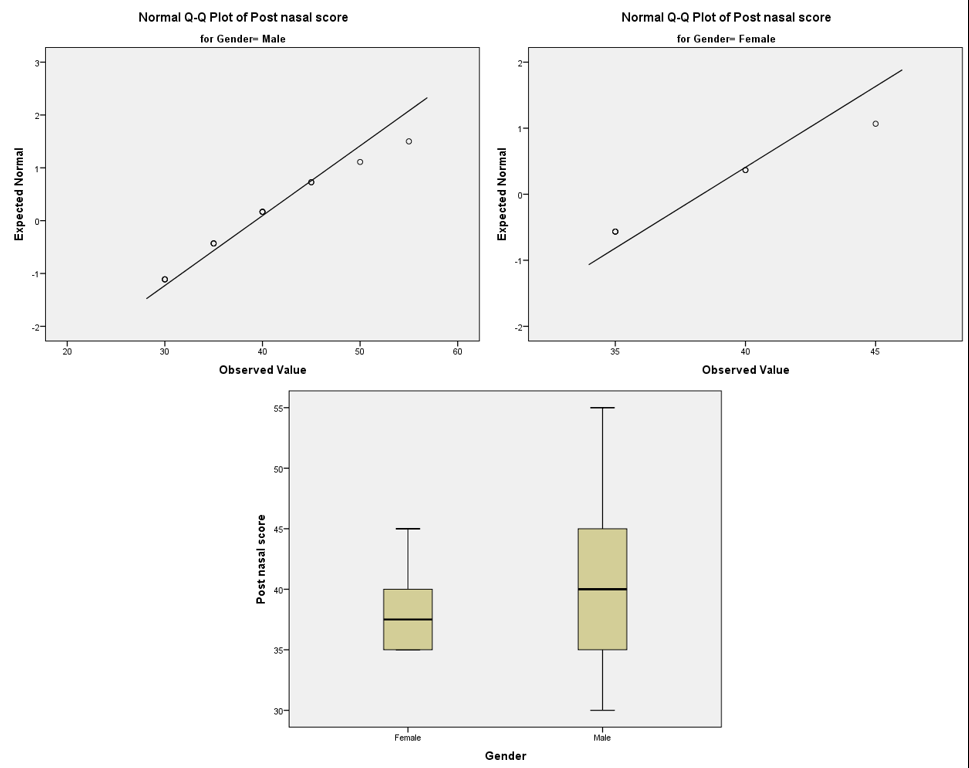


Supplementary Figure 2. Correlation between postoperative INV area on left side and postoperative nasal score.


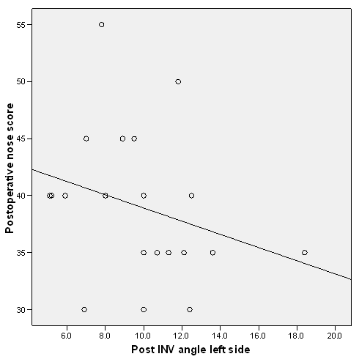


Supplementary Table 1. NOSE scores scale quality of life assessment questionnaire.

|  | Not a problem | Very mild problem | Moderate problem | Fairly bad problem | Sever problem |
| --- | --- | --- | --- | --- | --- |
| Nasal congestion or stiffness | 0 | 1 | 2 | 3 | 4 |
| Nasal blockage or obstruction | 0 | 1 | 2 | 3 | 4 |
| Trouble breathing through nose | 0 | 1 | 2 | 3 | 4 |
| Trouble sleeping | 0 | 1 | 2 | 3 | 4 |
| Unable to get enough air through nose during exercise or exertion | 0 | 1 | 2 | 3 | 4 |
